# Cyberincivility Experience of Korean Clinical Nurses in the Workplace: A Qualitative Content Analysis

**DOI:** 10.3390/ijerph17239052

**Published:** 2020-12-04

**Authors:** Sang Suk Kim, Ho Jeong Song, Jung Jae Lee

**Affiliations:** 1 Red Cross College of Nursing, Chung-Ang University, Seoul 06974, Korea; ssongho@gmail.com; 2 School of Nursing, University of Hong Kong, Hong Kong, China; leejay@hku.hk

**Keywords:** clinical nurse, experience, cyberincivility, qualitative study

## Abstract

Although clinical nurses use online platforms to acquire health-related information and communicate with other healthcare providers, there are increasing reports on their incivility exposure in cyberspace. However, an in-depth understanding of their cyberincivility experience is lacking. This study aimed to identify Korean clinical nurses’ perception and experience of cyberincivility. A qualitative study was conducted. Twenty clinical nurses from seven private and public hospitals in the Seoul metropolitan area were recruited using purposive sampling. Individual semi-structured interviews were conducted with the nurses from June to September 2019. Conventional content analysis was applied for the interview data analysis. Clinical nurses perceived cyberincivility as disrespectful and condemning behavior as users hide under the shield of anonymity to persecute others without fear of retribution. Four themes regarding participants’ cyberincivility experience emerged: unprofessional behavior, hierarchical communication, lack of respect and morality, and forming an inefficient work environment. The results of this study provide an understanding regarding clinical nurses’ experience of cyberincivility that goes beyond that of previous studies, which mainly focused on students. These results could increase awareness of cyberincivility among clinical nurses, and provide key information for the design of cybercivility educational programs and guidelines to curb cyberincivility, nurture professional online communication, and consequently improve quality of care.

## 1. Introduction

Online platforms have transformed the way people communicate in their daily lives. With over 1.8 million service users, these internet-based platforms, including social media, have gained particular popularity to primarily communicate with families, friends, and colleagues [1,2]. Online platforms have also played a significant role in communicating quality health information to achieve effective public health education [3]. Healthcare providers, including nurses, doctors, and pharmacists, use online spaces, including social media, to search for and share information. They also build relationships with other healthcare providers through these platforms [4,5].

Nurses spend significant time using online platforms to search for health-related information. Approximately 89 to 93% of the nurses in the United States of America (USA) use social media [4]. In South Korea (hereafter Korea), 98.7% of nursing students use online messaging applications, and 83.1% use Facebook, 46.1% used web portals, 15.9% use blogs, and 8.3% use Twitter [6]. Considering the fact that the use of social media services by the Korean population (48.2% in 2018) is consistently increasing [7,8], it can be deduced that the use of social media among nurses in Korea must have increased as well.

Despite the advantages of the internet, negative influences from online platform use have also been reported, including cyberbullying and cyberincivility [2,9,10]. Cyberbullying is a form of harassment that is repetitive and intentional, targeting vulnerable individuals, whereas cyberincivility is not necessarily intentional and repetitive [11,12]. Cyberincivility is defined as harmful, disrespectful, and insensitive behaviors that interfere with personal, professional, and social well-being in a cyber environment, violating personal relationships directly and indirectly [2]. These behaviors are regarded as deviant from the accepted standards or values held by most members of society [13]. This perforates in the form of insulting remarks, actions such as not responding to emails, workplace conduct, perception of supervisors, work performance, lowered productivity, and negative effects such as resignation, lowered self-esteem, depression, and sleep disturbance [10]. Cyberincivility may cause more psychological distress and anxiety than face-to-face incivility due to the public nature of online platforms and their ubiquitous and high accessibility [14]. Clinical nurses closely communicate with other nurses and healthcare providers using online platforms to provide patient care. Cyberincivility in the healthcare environment is perceived as a moderate to serious problem [8,15]. According to an integrative review, disclosing patient information, using derogatory language, and anonymous condemnation of patients or colleagues among healthcare providers including nurses were frequently observed in the clinical environment [16]. In a study that analyzed nurses’ and nursing students’ Twitter use [2], profanity, product promotion, sexual content, deprecation of patients, rude comments, attacking of other professions, and racial discrimination were commonly found as well. As such, nurses’ cyberincivility are frequently observed in clinical environments and their daily lives, and their attitude and behavior toward cyberincivility can affect their productivity in delivering nursing care and their compliance with medical ethics [2]. Therefore, it is imperative to develop educational and strategic guidelines to nurture acceptable attitudes and behavior in online spaces.

However, a majority of the previous studies on cyberincivility have only focused on students [15,16,17,18], and there is limited research that explored nurses’ experience of cyberincivility. Particularly, to our best knowledge, there are no studies that have explored Asian nurses’ experiences of cyberincivility, notwithstanding that sociocultural factors often shape the characteristics of cyberincivility [9,19].

The aim of this study was to explore Korean clinical nurses’ perceptions and experiences of cyberincivility.

## 2. Materials and Methods

### 2.1. Research Design

As there is a limited understanding of Asian nurses’ experiences of cyberincivility, a qualitative study using conventional content analysis [20] was conducted to explore the properties and patterns of clinical nurses’ cyberincivility experiences. Conventional content analysis is commonly used when the understanding of the research phenomenon is lacking in existing studies [20]. The study followed the Consolidated criteria for reporting qualitative research (COREQ) checklist [21].

### 2.2. Research Participants

Using purposive and snowball sampling, registered nurses who worked in clinical contexts, such as private or public hospitals in the Seoul metropolitan area, were recruited for this study. The eligible participants were as follows: registered nurses who (1) worked full-time, (2) communicated and interacted with other healthcare providers through emails and social media services, and (3) had experienced cyberincivility in the past 30 days. A total of 20 nurses were recruited for this study as data saturation was attained.

### 2.3. Data Collection

Individual in-depth interviews to study the experiences related to cyberincivility were conducted by a researcher using a semi-structured questionnaire with open-ended questions from June to September 2019. The participants’ demographic characteristics and general details were collated before conducting the interviews.

Using an interview guide (Table 1), the interviews were conducted in a meeting room at the hospital where the participants were employed or a quiet cafe where the participants would feel comfortable in a separated space. With consent, the interviews were recorded, and reflective notes were taken during the interviews.

After each interview, the participant’s answers were summarized by the first author. The summary was used to update the interview guide and arrange follow-up interviews to gain an in-depth understanding as required. If follow-up face-to-face interviews could not be conducted due to their shift schedules, emails and video calls were used instead.

The first interviews with each participant lasted for approximately 30 to 50 min each, and the following interviews lasted for around 20 to 30 min. Eight participants were interviewed up to two or three times (one repeated interview for six nurses, two repeated interviews for two nurses).

### 2.4. Ethical Consideration

This study was conducted after obtaining approval from the Institutional Review Board of University (IRB NO: 1041078-201904-HRSB-117-01). Written informed consent was obtained from each participant.

### 2.5. Data Analysis

All interview recordings were transcribed verbatim. Conventional content analysis with an inductive approach was applied to the research data [20]. First, the interview transcripts and notes were read a few times to understand and identify the meanings of the participants’ experience from their perspective, therefore enabling the understanding of the overall meaning. Second, meaningful words, sentences, and paragraphs from the interview transcripts were extracted and coded. Third, the coded materials were grouped together according to their similarities and differences, and themes were identified based on similar codes. Fourth, themes with wider implications were developed according to the relevance of the previously deduced themes, and the relevance between the themes and grouped categories or subcategories was confirmed. Constant comparison is an analytic strategy to continuously compare the researcher’s emerging interpretations during data analysis with existing data and findings in order to enhance data analysis quality [22]. NVivo 12 software (QSR International; Melbourne, Australia) was used to manage the collected data during data analysis. After analysis, interview quotations in Korean that were selected for reporting were translated into English by a professional translator and a bilingual researcher (English and Korean) and then two bilingual researchers back-translated the same.

### 2.6. Trustworthiness

To ensure that the quality of research data is maintained, the evaluation criteria for qualitative research proposed by Guba and Lincoln [23], which are credibility, transferability, dependability, and confirmability, were considered for this study (Table 2).

## 3. Results

The characteristics of the 20 participants are presented in Table 3. All participants were female, and the mean age was 32.3 (SD ± 2.58) years. The average length of clinical experience was seven years and nine months (SD ± 3.24). Seventeen participants (85%) had one to five social media accounts, nine participants (45%) spent an average of less than an hour on social media, and 17 participants (85%) responded that the cyberincivility problem is either moderate or severe.

### 3.1. Definition of Cyberincivility by Clinical Nurses

Clinical nurses in this study communicated that “criticism, rudeness, harassment, plagiarism, belittling, disdain, disrespect, unethical, out of the norm, and disclosure of information” were the words that could be related to cyberincivility. They defined cyberincivility as a concept where one can insult others in online spaces where anonymity is guaranteed.

*When I heard the term cyberincivility, I first thought of how people make comments irresponsibly, don’t take other people’s words seriously, and are straightforward with words and do not communicate politely, largely because you can be anonymous online.*

(Participant 20)

### 3.2. Cyberincivility Experiences as Perceived by Clinical Nurses

Four themes, nine categories, and 23 subcategories were identified from the clinical nurses’ cyberincivility experience. The four themes were unprofessional behavior, hierarchical communication, lack of respect and morality, and forming an inefficient work environment.

#### 3.2.1. Theme 1: Unprofessional Behavior

Unprofessional behavior referred to the posting of content online by clinical nurses or professionals from other fields that does not comply with the code of ethics or code of conduct for healthcare providers. Certain posts made online by healthcare providers disparaged clinical nurses or professionals in other fields or formed a prejudice against patients by criticizing them, which resulted in direct or indirect harm to healthcare providers.

*YouTube is so popular these days. I’ve seen certain things shared there that should be protected, like patients’ personal information. The patient’s identity is not revealed, but it can be figured out easily because of the information shared. I wonder if it’s really okay to do that.*

(Participant 14)

All the participants were trained regarding the protection of patients’ privacy when they started working in the clinical setting; however, they had witnessed many instances where patients’ private information was leaked through social media. They recognized such behavior as cyberincivility and were concerned that it could be harmful to patients or their families.

*If people find out that their personal information has been disclosed on social media, it’ll definitely hurt their feelings. Those who have had their private information leaked are probably really shocked and disheartened. They would not trust any healthcare provider anymore as professionals and they’ll be more careful.*

(Participant 19)

Most of the participants had a negative attitude toward the online learning provided by their hospitals. They believed that skipping lectures or videos, having other colleagues watch lectures or videos in place of them, having another person take the test, or collaborating to pass the online tests were all considered as cyberincivility. When they were asked to evaluate the professor or instructor at the end, they used more direct and negative words as compared to evaluating offline classes. They used terms such as “boring” or “wish it was shorter” due to the anonymity.

*We often skipped the online learning that all the nurses in the hospital were required to take. In fact, sometimes we even had someone in the department who used computers constantly watch it for us.*

(Participant 12)

*As we did not have to physically be in a classroom, we could easily skip through the content. Also, while evaluating the learning we used terms like, “it’s boring” or “I wish it was shorter.” In fact, even I have given negative feedback.*

(Participant 20)

#### 3.2.2. Theme 2: Hierarchical Communication

Increased use of social media, including messaging applications and emails, which are the means of communication in a medical work environment, led to an increase in the experiences of cyberincivility. The participants pointed out that the hierarchical culture within the nursing community and the authoritative backdrop of the healthcare environment in Korea led to problems with communication even in online spaces. For example, if a nurse did not agree with another member of the department, especially with a senior nurse, or if the situation was unsatisfactory, the nurse would be publicly criticized online or ignored by halting communication. Such culture was observed in the relationship between nurses, between nurses and their supervisors, and between nurses and doctors.

*Head nurses or doctors sometimes force us to work [through messaging applications] and even ask for personal favors besides our work. Younger residents sometimes order senior nurses with more clinical experience than themselves around using a commanding tone.*

(Participant 4)

Institution-wide messaging applications or group chatrooms on social media were used to share ward members’ mistakes or shameful issues in the hospital, and members were publicly criticized or rebuked.

*One senior nurse took a picture [of my mistake] and posted it on the group chatroom saying, “There is someone who still does this,” so that everybody could see it. It was my fault, but I was still really offended. Posting something on a group chatroom publicly is rude and not an appropriate way to correct someone who has that much experience.*

(Participant 8)

#### 3.2.3. Theme 3: Lack of Respect and Morality

Nursing tasks are carried out based on the cooperation among various departments, hence effective communication between these departments is crucial. However, the participants responded that most departments did not understand the roles and tasks of other departments. Additionally, as they are overwhelmed with work, most individuals persisted with their opinions and expressed their emotions strongly through online communication.

*Everyone is busy with their work and they tend to become emotional, but these feelings are not controlled and are expressed explicitly in online spaces. Whoever is on the other side gets upset too.*

(Participant 20)

When such cyberincivility occurred where mutual respect is not taken into consideration, it eventually results in reduced trust between all individuals. Working with other healthcare providers without mutual trust increased workload as individuals doubted each other’s work due to concerns regarding medical malpractice and shifting of responsibility. This required additional confirmation processes and worsened the working condition.

*Communication between nurses and other healthcare providers won’t be smooth and trust will not be built [due to cyberincivility]. We have to repeatedly confirm if the work has been done due to a lack of trust, and this is mentally exhausting. I believe that this is the biggest problem.*

(Participant 9)

The participants responded that profanity, exaggerated emotions, provocative comments, and false information were posted online as the person posting these could remain anonymous and such interactions were not face-to-face.

*As these interactions do not happen face-to-face, people can easily get away with them. You can’t really say these things to someone’s face, but when online (cyberincivility), it is much easier because you don’t know who you are talking to and you don’t know anything about them.*

(Participant 3)

#### 3.2.4. Theme 4: Forming Inefficient Work Environment

The experience of cyberincivility worsened interpersonal relationships, including those between different professions. Misunderstandings between professionals interfered with the cooperation among healthcare providers and led to prejudice against other professions.

*Even though they are colleagues who I have to work with, the relationship that’s been ruined online due to cyberincivility is reflected in real life as well.*

(Participant 16)

*We easily misunderstand people in other professions. However, a lot of our work involves cooperating with them, therefore this makes things difficult by pushing everyone away from each other.*

(Participant 7)

The participants stated that the nurses who were hurt emotionally due to cyberincivility often developed depression, which in turn resulted in a passive attitude when caring for patients. These cyberincivility instances ultimately had a negative impact on the patient–nurse relationship.

*We feel horrible because of the impact of cyberincivility. The negative effect on our nursing work. As a result, instead of genuinely caring for patients, sometimes it just feels like a part of work and seems troublesome. I can’t greet the patients nicely when they call me, and I sometimes even get annoyed.*

(Participant 7)

The participants reported that being frequently contacted outside of working hours through social media increased work stress and emotional burden, causing exhaustion. Nurses who were unfairly overloaded with work suffered from low morale and felt discouraged. This eventually led them to consider resigning from the job.

*Telling us to work harder through messaging applications even after our working hours and randomly sending out unnecessary notices leads to more emotional burden, over and above the work itself. [Because of that] the work was overwhelming, and I got burnt out and eventually thought about quitting.* (Participant 4)

## 4. Discussion

This study identified that clinical nurses in Korea experienced cyberincivility in their online communication with other nurses and healthcare providers under four major themes; unprofessional behavior, hierarchical communication, lack of respect and morality, and forming an inefficient work environment.

Clinical nurses in this study perceived cyberincivility as disrespectful and condemning behavior based on anonymity, which is in concordance with definitions from previous studies [9,10,16]. The words frequently used to describe cyberincivility were criticism, rudeness, disregard, disrespect, vicious comments, unethical, and out of the norm, which implies that such behavior or conduct is considered inappropriate by nurses, doctors, or professionals in the workplace. This can be inferred from the similarities in clinical and educational environments reported by clinical nurses who were the subjects for this study and health professional students who were the subjects in previous research [9,10,16]. By observing health professionals’ professional behavior and attitudes during clinical practice, health professional students would experience cyberincivility directly or indirectly during college life and therefore consider aggression toward healthcare providers and patients in online spaces as unprofessional conduct. From this perspective, providing cybercivility education in universities would greatly contribute toward preparing these students as future clinical experts by equipping them with appropriate behaviors, attitudes, and responses toward cyberincivility.

Participants recognized negative opinions shared online about fellow healthcare professionals and patients as “unprofessional behavior,” corresponding with studies [3] that reported negative expressions disparaging colleagues or organizations, as well as patient information disclosures, constitute cyberincivility. According to previous studies, cyberincivility behaviors regarding online learning included ambivalence toward online discussions and cheating on online quizzes, similar to participants in this study using stand-ins to avoid hospital-mandated nursing in-service education, which ultimately hindered learning and affected social health [15,16,24]. Participants providing unconstructive feedback to online learning under the shield of anonymity is also a form of cyberincivility. Previous research has shown that the tendency to indulge in cyberincivility increases as anonymity is strengthened [25] and certain behaviors are performed when it is considered beneficial [26]. The ability to express negativity and humiliate others without having to bear the consequences can therefore be viewed as the benefit of and perpetuating factor for cyberincivility. However, cyberincivility can become a legal problem and the poster can be traced and exposed [27] resulting in financial harm and reputational damage [27,28]. There is evidence that while unprofessional behavior may first have a negative impact on the individual, it can go on to impact the institution and, finally, the quality of the services provided [29]. Regular provision of in-service education on cyberincivility and professionalism with examples of potential legal issues should be provided to all healthcare professionals [30]. The establishment of related workplace policies regarding cyberincivility and professional behavior will also be helpful. To increase engagement regarding cyberincivility learning for clinical nurses, a diverse range of educational topics to increase realism should be incorporated, with the use of more animated and interesting delivery strategies such as virtual reality.

Under “inappropriate communication”, participants reported that hierarchical communication was prevalent with an authoritative culture in hospitals, and messaging applications were used to criticize healthcare providers publicly or for sharing confidential information in the hospital. This is similar to a Korean study that reported a hierarchical communication structure between senior nurses and doctors in operating rooms, creating an environment that made nurses feel uncomfortable in voicing their opinions [31]. A hierarchical organizational culture entailing top-down command or a control-based approach is considered common in workplaces in Korea [32,33], and clinical nurses considered that learning to communicate accordingly was essential for assimilating into such workplace cultures [32]. Consequently, nurses can feel oppressed and forced to work, exacerbating their psychological burdens, creating peer conflict, and hindering effective communication within the institution [34] and result in online criticism of vertical organizational structures. The flattening of hierarchies in organizational culture reforms is thus essential to increase nurses’ performance and productivity [30]. Surveys on workplace-related experiences can be conducted, with facilitated discussions focusing on effective interprofessional and intraprofessional communication, while encouraging the sharing of opinions [32,35] and adopting strategies for civility and incivility awareness

According to the third theme of this study, “lack of respect and morality”, clinical nurses noted a lack of understanding between healthcare professions, aggravated by their own overwhelming workloads, led to instances of cyberincivility that damaged mutual trust. A previous study found that cyberincivility was associated with increased workloads [36]. Administrators should ensure that clinical nurses are treated equally in the operational, distributional, and interpersonal dimensions of powerful organizational cultures and make efforts to improve nurses’ job satisfaction [30].

“Forming an inefficient work environment” emerged as a final theme in this study. The clinical nurses responded that the relationship between nurses and patients and among professionals in other fields worsened due to cyberincivility, and consequently, mutual cooperation became difficult. Similar to the findings from another study, the communication between nurses and other professionals was difficult due to a lack of mutual respect, the egotism of different professions, and a lack of understanding of others’ responsibilities [37]. Inefficient communication between nurses and doctors causes frequent conflicts and leads to hostility between them, and nurses lose self-confidence while feeling intimidated, angry, and upset, thereby reducing the quality of medical service and nursing care [38]. A positive and powerful relationship between healthcare providers is related to job satisfaction [39] and positively influences patient outcomes [40]. The success of hospitals and the effectiveness of the services they provide are highly dependent on effective communication within the institution, acceptance of the organization’s culture, and cooperation [30]. A response protocol, training to appropriately handle cyberincivility experiences based on case studies [2], a consultation or counseling service system for psychological problems resulting from cyberincivility experiences may reduce the incidence of cyberincivility.

This study has several limitations as well. As the participants were recruited from the Seoul metropolitan area, their experience of cyberincivility may be different from that of nurses in other regions of Korea or in other Eastern Asian countries that have a similar social and workplace culture (e.g., China and Japan). Therefore, further studies using quantitative research designs with larger samples will be useful to test the findings from this study. The participants were all females therefore cyberincivility experiences from the perspective of male nurses were not examined, limiting generalization and application to the entire group of clinical nurses. The experience of cyberincivility between nurses and individuals in other professions was reported from the nurses’ perspectives in this study. Therefore, examining the experience of healthcare providers in other professions can also deepen the understanding of cyberincivility experiences of clinical nurses. Despite these limitations, this study went beyond the scope of previous studies that have investigated cyberincivility among adolescents or students in nursing or other medical fields. It is significant as it examined the patterns based on which the clinical nurses experience cyberincivility in hospitals, where mutual cooperation and communication are crucial.

## 5. Conclusions

The study provided an in-depth understanding of clinical nurses’ experience of cyberincivility in the workplace. Considering the lack of interventions and guidelines addressing cyberincivility among clinical nurses, the results of this study can inform researchers and policymakers in developing appropriate interventions and guidelines. The findings can also increase nurses’ awareness so that they can maintain professionalism in cyberspace and real-life clinical settings. As the study specifically targeted clinical nurses, the results can also provide a vital foundation for the development of education programs on cyberincivility within the healthcare environment to curb cyberincivility and nurture professional online communication. Efforts to prevent and approach cyberincivility issues, the efficiency of interprofessional communication, and cooperation may improve cyberincivility and lead to improved patient care.

## Figures and Tables

**Table 1:** Interview guide (initial version).

**Opening Questions**
What comes to your mind when you hear the word cyberincivility in online spaces such as social media, email, and online education?
How do you define cyberincivility in online spaces such as social media, email, and online education?
**Focused Questions**
What kind of workplace incivility have you experienced from colleagues, professors, or other healthcare providers?
Why do you think cyberincivility happens in your work or education environment?
What impact does cyberincivility (official or unofficial) have on your work or education environment?
As a healthcare provider, what are your responsibilities for the posts or information (contents) that you share on the internet, if there are any?
What problems may arise among nurses or between different professions due to cyberincivility?
**Ending Question**
Are there any other comments that you would like to make?

**Table 2:** Trustworthiness in the study.

Criteria	Method
Credibility	Follow-up interviews were conducted after the first interview to accurately determine the meaning for increased credibility. Participants’ nonverbal behaviors during the interview were also transcribed. To confirm if the interview details were transcribed correctly, the transcription was shown to the participant to verify the intended meaning before analyzing the data.
Transferability	Clinical nurses from multiple hospitals were recruited instead of from one hospital to ensure the transferability of the study. The data were collected until saturation and the stage that no new information was found in the interview notes and transcripts. Accordingly, various experiences were examined and the suitability increased through in-depth analysis.
Dependability	To secure consistency, relevant research and literature reviews were sufficiently collected and reviewed in detail. The research procedure and analysis methods were designed on the advice of two researchers with extensive experience in qualitative research. Furthermore, several rounds of revisions were made to ensure consistent research results.
Confirmability	The study findings and the researchers’ interpretation were sent to the participants to seek their confirmation. In addition, the findings were also sent to two experts in cyberincivility research to obtain their feedback.

**Table 3:** General characteristics of the participants.

Variable	Option	N (%) or M ± SD
Sex	Female	20 (100)
Age (years)		32.3 ± 2.58
Education	Undergraduate	17 (85)
	Postgraduate	3 (15)
Length of clinical experience (years)	Across all wards	7.9 ± 3.24
	In the current ward	5.6 ± 3.56
Number of social media accounts	1–5	17 (85)
	6–10	2 (10)
	11–20	1 (5)
Time spent using social media per day	Less than 1 h	9 (45)
	1–3 h	4 (20)
	4–6 h	5 (25)
	Over than 7 h	2 (10)
Number of contacts	10–50	3 (15)
	51–100	6 (30)
	101–200	7 (35)
	201–500	4 (20)
Number of emails received per day	0–10	12 (60)
	11–20	7 (35)
	21–50	1 (5)
Number of text messages received per day	Less than 20	3 (15)
	21~50	9 (45)
	51~100	2 (10)
	101 or more	6 (30)
Perception of cyberincivility	Mild problem	3 (15)
	Moderate problem	2 (10)
	Severe problem	15 (75)
